# Genome plasticity of *Vibrio parahaemolyticus*: microevolution of the 'pandemic group'

**DOI:** 10.1186/1471-2164-9-570

**Published:** 2008-11-28

**Authors:** Haihong Han, Hin-chung Wong, Biao Kan, Zhaobiao Guo, Xiaotao Zeng, Shengjun Yin, Xiumei Liu, Ruifu Yang, Dongsheng Zhou

**Affiliations:** 1 State Key Laboratory of Pathogen and Biosecurity, Beijing Institute of Microbiology and Epidemiology, Beijing 100071, PR China; 2 Institute of Nutrition and Food Safety, Chinese Center for Disease Control and Prevention, Beijing, 100050, PR China; 3 Department of Microbiology, Soochow University, Taipei, Taiwan, PR China; 4 Institute for Communicable Disease Control and Prevention, Chinese Center for Disease Control and Prevention, Beijing, 102206, PR China

## Abstract

**Background:**

Outbreak of *V. parahaemolyticus *infections occurred since 1996 was linked to a proposed clonal complex, the pandemic group. The whole genome sequence provides an unprecedented opportunity for dissecting genome plasticity and phylogeny of the populations of *V. parahaemolyticus*. In the present work, a whole-genome cDNA microarray was constructed to compare the genomic contents of a collection of 174 strains of *V. parahaemolyticus*.

**Results:**

Genes that present variably in the genome accounted for about 22% of the whole gene pool on the genome. The phylogenetic analysis of microarray data generated a minimum spanning tree that depicted the phylogenetic structure of the 174 strains. Strains were assigned into five complexes (C1 to C5), and those in each complex were related genetically and phylogenetically. C3 and C4 represented highly virulent clinical clones. C2 and C3 constituted two different clonal complexes 'old-O3:K6 clone' and 'pandemic clone', respectively. C3 included all the 39 pandemic strains tested (*trh*^-^, *tdh*^+ ^and GS-PCR^+^), while C2 contained 12 pre-1996 'old' O3:K6 strains (*trh*^+^, *tdh*^- ^and GS-PCR^-^) tested herein. The pandemic clone (post-1996 'new' O3:K6 and its derivates O4:K68, O1:K25, O1:KUT and O6:K18) might be emerged from the old-O3:K6 clone, which was promoted by acquisition of *toxRS*/new sequence and genomic islands. A phylogenetic intermediate O3:K6 clade (*trh*^-^, *tdh*^- ^and GS-PCR^+^) was identified between the pandemic and old-O3:K6 clones.

**Conclusion:**

A comprehensive overview of genomic contents in a large collection of global isolates from the microarray-based comparative genomic hybridization data enabled us to construct a phylogenetic structure of *V. parahaemolyticus *and an evolutionary history of the pandemic group (clone) of this pathogen.

## Background

*Vibrio parahaemolyticus *is a halophilic, Gram-negative bacterium. As a natural inhabitant of estuarine marine water, it is widely distributed in seawater and sediments, or frequently associated with marine shellfish. It is the leading cause of human food poisoning caused by consumption of the contaminated seafood, especially raw seafood such as oyster, throughout the world.

In contrast to most environmental isolates, clinical *V. parahaemolyticus *is often able to produce thermostable direct haemolysin (TDH) and/or TDH-related toxin (TRH), encoded by the *tdh *and *trh *genes, respectively [1]. However, clinical isolates in absence of both *tdh *and *trh *have been identified [2]. In addition to TDH and TRH, virulence-related determinants still include thermolabile haemolysin (encoded by the *tl *gene), two type III secretion systems, and the ability of adhesion and invasion of enterocytes [1,3,4]. Clinical *V. parahaemolyticus *is often characterized as Kanagawa phenomenon (KP) positive by exhibiting β-haemolysis on the Wagatsuma agar due to the production of TDH [3].

Serotyping based on O and K antigens can differentiate isolates of *V. parahaemolyticus*, and accordingly 13 O groups and 71 K types are identified by using the commercial antisera. Traditional molecular typing studies based on pulsed-field gel electrophoresis (PFGE), arbitrarily primed PCR (AP-PCR) and multi-locus sequence typing (MLST) have been employed to distinguish among isolates [5-9].

Outbreaks of *V. parahaemolyticus *infections occurred since 1996 were initially linked to a predominant serovar O3:K6 (*tdh*^+ ^and *trh*^-^). This 'new' O3:K6 appeared firstly in the February of 1996 in India, and then rapidly spread worldwide, particularly in coastal countries and regions [10-12]. The PFGE, AP-PCR and MLST studies [5-9] revealed that the new O3:K6 and its derivates O4:K68, O1:K25 and O1:KUT isolated since 1996 gave very similar fingerprint patterns (FPs) or sequence types (STs), suggesting that they constitute a clonal complex. These strains are collectively called the 'pandemic group' that is thought to be responsible for the pandemic outbreaks [10-12].

The pandemic group possesses a variety of 'unique' DNA markers, including *toxRS*/new sequence (GS-PCR) [10,12], ORF8 in the phage f237 [13,14], an insertion sequence within the Hu-α gene (Hu-α/insertion) [15], a 930 bp AP-PCR fragment (PGS-PCR) [16], and an open reading frame VP2905 [17]. PCR methods for detection of these markers have been developed accordingly for distinguishing the pandemic group from other *V. parahaemolyticus *strains. However, further studies indicated none of the first three markers were specific to the pandemic group [12,18]. Notwithstanding, a positive detection of both *tdh *and *toxRS*/new sequence by PCR (*tdh*^+ ^and GS-PCR^+^) can reliably identify the pandemic strains [12,18]. The *toxRS*-targeted GS-PCR is based on the observation that the pandemic strains have a unique sequence (namely *toxRS*/new sequence) within the *toxRS *operon that encodes transmembrane proteins [10,12].

The complete genome sequences of a pandemic O3:K6 strain RIMD2210633 [19] and a non-pandemic O3:K6 strain AQ3810 have been determined [20]. The genome of strain RIMD2210633 consists of two circular chromosomes of 3,288,558 bp and 1,877,211 bp, and it harbors 4832 coding sequences (genes). The whole genome sequence provides an unprecedented opportunity for illustrating genome plasticity and phylogeny of *V. parahaemolyticus *populations. In the present work, the genome dynamics within 174 strains of *V. parahaemolyticus*, due to gene acquisition/loss, was determined by microarray-based comparative genomic hybridization (M-CGH). Subsequent clustering and phylogenetic analysis outlined a phylogenetic structure of *V. parahaemolyticus *as well as an evolutionary history of the pandemic group.

## Results and discussion

### Strain collection

The 174 strains of *V. parahaemolyticus *[see Additional file 1] used in this study include 125 clinical isolates and 49 non-clinical ones. The non-clinical strains were isolated either from seafood or from marine environments. In a previous study [9], a collection of 535 strains of *V. parahaemolyticus *were analyzed by PFGE, generating 115 PFGE patterns. Accordingly, 129 strains covering almost all the PFGE patterns were picked out for this study. In addition, 42 strains from six provinces (Guangxi, Hebei, Inner Mongolia, Niaoning, Shandong, and Shanghai) in China and three international isolates were added into the strain collection. All together, the 174 strains came from 13 countries, namely China (Mainland, Hong Kong and Taiwan), Bangladesh, India, Indonesia, Japan, Korea, Malaysia, Maldives, Phillipine, Singapore, Spain, Thailand, and United States. They were isolated between 1951 and 2007, with about 15% of them being isolated before 1990. These strains should represent the sufficient abundance of *V. parahaemolyticus *populations.

### Basic features of bacterial strains

**Figure 1:**
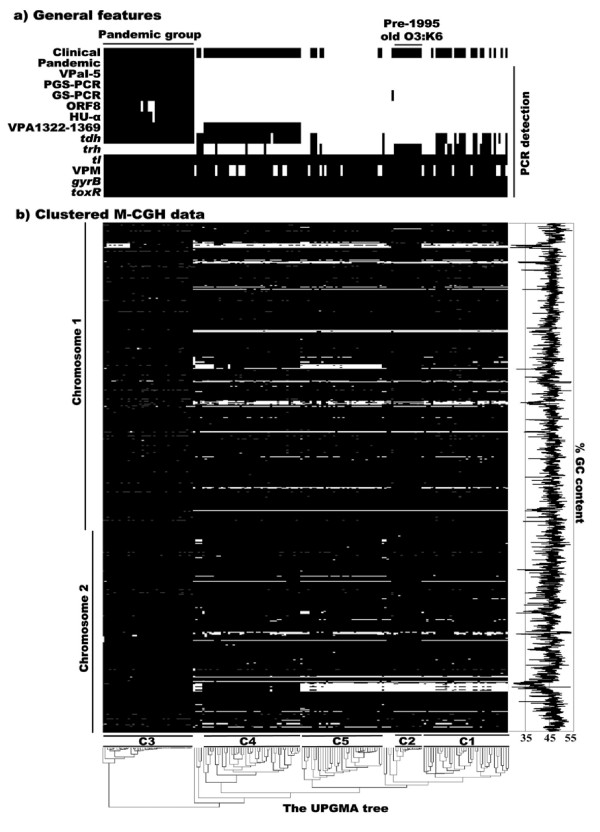
**Gene distribution based on M-CGH and PCR**. a) PCR-based characterization of the 174 strains. b) Clustered M-CGH data by the UPGMA method. Each column represents a strain, while each row stands for a different gene. Genes are arranged according to the genomic location of strain RIMD2210633. For each strain, a black area indicates the presence of a gene, whereas white absence, and grey missing data. The GC content curve is shown on the right. The UPGMA tree is shown at bottom.

By using the primers described previously [see Additional file 2], PCR was performed to screen the presence of various DNA markers, including *toxRS*/new sequence (GS-PCR) [10,12], ORF8 [13], Hu-α/insertion [15], PGS-PCR sequence [16], *V. parahaemolyticus*-specific sequences of VPM [21], *gyrB *[22] and *toxR *[23], and the *tdh*, *trh *and *tl *genes. This analysis was able to assess the reliability of corresponding PCR-based detection/identification, and also dissect the basic genetic features of *V. parahaemolyticus *strains tested (Figure 1). The first four markers have been proposed for identification of pandemic strains [10,12,13,15,16]. We defined the pandemic group based on the previous genotypic definition (GS-PCR^+^, *tdh*^+ ^and *trh*^-^) [9,12]. Accordingly, 39 strains were identified to be the pandemic group members. Notably, in addition to the O3:K6, O4:K68, O1:K25 and O1:KUT strains, one O6:K18 strain was also assigned into this group.

In addition to the 39 pandemic strains, a *tdh*^- ^O3:K6 strain S093 gave the positive GS-PCR reaction. Three of the 39 pandemic strains were negative for ORF8; one of these three was negative for Hu-α/insertion. Thus, none of *toxRS*/new sequence, ORF8 and Hu-α/insertion was reliable for PCR-based identification of the pandemic strains, which confirmed the previous results [9,12,24]. The positive PGS-PCR was detected only for the 39 pandemic strains, indicating that the corresponding DNA marker (namely the PGS-PCR sequence) was specific to the pandemic group. In addition, we confirmed that the gene cluster VP2900-2910, constituting a genomic island named VpaI-5 [25], was a unique genomic feature of the pandemic group (see below).

*V. parahaemolyticus*-specific sequences of *gyrB *[22], *toxR *[23] and VPM [21] have been characterized for the PCR identification of this bacterium at the species level. Here, the first two sequences were detected in all the 174 strains, giving an inclusivity of 100%. However, a total of 25 strains gave negative PCR results for VPM, with an inclusivity of only 86%; thereby, this marker was not reliable for the bacterial identification.

The *tl *gene was universally present in the 174 strains tested. The universal presence of this gene in *V. parahaemolyticus *strains has been characterized previously [26]. Despite the 100% correct identification of *V. parahaemolyticus *by the *tl *gene-based PCR, the test was lack of the specificity, i.e., false-positive PCR results could be seen in other *Vibiro *species [26].

94% of the clinical strains harbored the *tdh *and/or *trh *genes; especially, the *tdh*^+ ^ones constituted 90% of the clinical strains [see Additional file 3]. In contrast, 90% of non-clinical strains contained none of the two genes. These data supported the notion that the presence of *tdh *and/or *trh *was closely correlated with the pathogenicity of *V. parahaemolyticus*.

### Efficacy of M-CGH methodology

In our previous works, a *Yersinia pestis *DNA microarray was employed to compare the genomic content of *Yersinia *strains [27], promoting us to establish the standard operation procedures of M-CGH. The efficacy of M-CGH in the present work was assessed by the control hybridizations of 'Reference DNA versus Reference DNA'. After the data filtering procedures, all the genes gave a correct prediction of their presence. The control hybridizations still include 'genomic DNA of strain S004 versus Reference DNA'. The gene cluster VP2900-2910 was shown to be absent in this strain, as determined by our preliminary PCR experiments (data not shown). The microarray analysis confirmed the absence of VP2900-2910 in this strain. A total of 4021 genes were included in the final microarray dataset that contained the M-CGH profiles of the 174 strains. Genes were categorized as either present (1), absent (0) or missing data for each strain.

### Phylogeny

**Figure 2:**
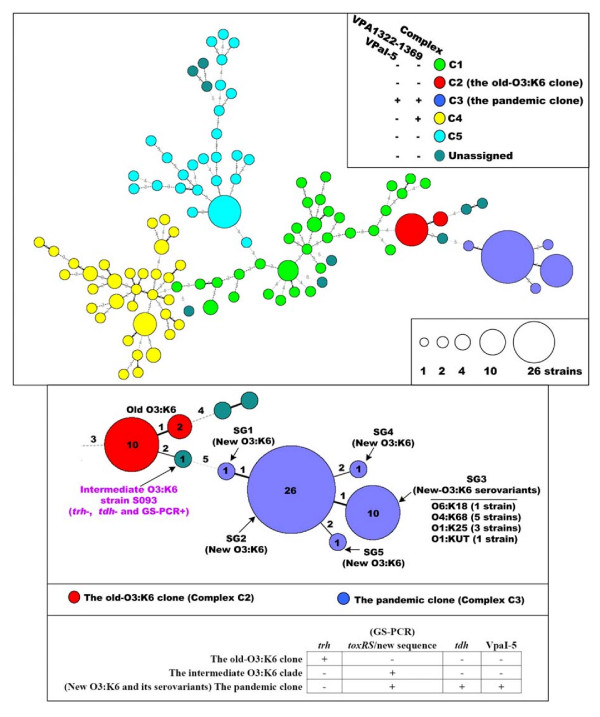
**Minimum spanning tree of 174 global strains**. Each circle indicates a haplotype (node), and a larger size of the circle corresponds to a larger number of strains included. The number along each edge reflects the phylogenic distance between each two neighboring nodes. In addition, a thicker edge corresponds to a shorter phylogenic distance. 165 (98 nodes) of the 174 strains are assigned into five complexes, C1 to C5, while the remaining nine strains (nine nodes) are indicated as 'unassigned'. Nodes in different complex are labeled with different colors. The 39 pandemic strains are assigned into five sub-groups, SG1 to SG5 (different SGs distribute in different nodes). The presence or absence of *trh*, *tdh*, *toxRS*/new sequence and VpaI-5 in various groups of strain are shown as well.

We performed the clustering analysis on the binary M-CGH data by the UPGMA method (Figure 1). This analysis generated a similarity matrix as well as a UPGMA tree (Figure 1). On the basis of the similarity matrix, a minimum spanning tree was constructed to give a phylogenetic structure of the 174 strains (Figure 2). Based on the minimum spanning tree as well as the UPGMA tree, 165 of the 174 strains were assigned into five complexes, C1 to C5. Each complex, in particular C2 and C3, showed a considerable conservation of composition/number of genes that present variably in the genome (variably-presented genes, VPGs) (Figure 1). This demonstrated that strains in each complex were related genetically and phylogenetically.

General features of strains in different complexes were shown in Table 1. All the strains in C2 to C4 were clinical. C3 included all the 39 pandemic strains tested (*trh*^- ^and *tdh*^+^), while C2 contained 12 old O3:K6 strains (*trh*^+ ^and *tdh*^-^) used in this study. The number of VPGs in C2 and C3 was obviously lower than that in all the other strains. An extremely high conservation of genomic content was observed in these two groups of strains (Figure 1), therefore, constituting two different clonal complexes, namely 'old-O3:K6 clone' (C2) and 'pandemic clone' (C3).

**Table 1:** Features of strains of different complexes

		% Percentage (number of strains)						
		
Complex	Number of strains	Clinical	Pandemic	VPaI-5^+^	PGS-PCR^+^	VPA1322-1369^+^	*tdh*^+^	*trh*^+^
		59 (22)	0 (0)	0 (0)	0 (0)	0 (0)	49(18)	54 (20)
C2	12	100 (12)	0 (0)	0 (0)	0 (0)	0 (0)	0 (0)	100 (12)
C3	39	100 (39)	100 (39)	100 (39)	100 (39)	100 (39)	100 (39)	0 (0)
C4	42	100 (42)	0 (0)	0 (0)	0 (0)	100 (42)	98 (41)	7 (3)
C5	35	17 (7)	0 (0)	0 (0)	0 (0)	0 (0)	9 (3)	11 (4)
Unassigned	9	33 (3)	0 (0)	0 (0)	0 (0)	0 (0)	44 (4)	44 (4)

Total	174	72 (125)	22 (39)	22 (39)	22 (39)	47 (81)	60 (105)	25 (43)

C2: the old-O3:K6 clone; C3: the pandemic clone.

VPaI-5 (see below) was unique to the pandemic clone (C3) according to the M-CGH data. Genes in VPaI-5 were further screened in the 174 strains by PCR [see Additional file 2], confirming the M-CGH results. Accordingly, two pandemic-group-specific markers, PGS-PCR sequence and VPaI-5, were identified (Figure 1).

The *tdh *gene was present in all the C3 strains and most (98%) of the C4 strains, but not in the C2 ones. According to the M-CGH data, the gene cluster VPA1322-1369 was found in all the C3 and C4 strains, but not in all the other strains, which was further confirmed by PCR screening [see Additional file 2]. This gene cluster encodes a type III secretion system (T3SS) that contributes to the virulence of *V. parahaemolyticus *[4]. Both VPA1322-1369 and *tdh *were harbored in a pathogenicity island VPaI-7 (see below) in strain RIMD2210633. The presence of both *tdh *and VPA1322-1369 in almost all the C3 and C4 strains suggested that these strains might represent the highly virulent clones. Indeed, the C3 strains (the pandemic group) has been linked to the outbreaks of *V. parahaemolyticus *infections since 1996.

Both *trh *and *tdh *were absent from 31 of the 35 strains in the C5 complex. 83% (28 of the 35 strains) of the C5 strains were non-clinical, and all these 28 strains were negative for both *trh *and *tdh*. Thus, the C5 complex is linked to environmental isolates. Lack of both *trh *and *tdh *does not impede *V. parahaemolyticus *to be non-virulent, due to the existence of other virulence factors (e.g. type III secretion system) [4]. Animal virulence experiments on a collection of typical C5 strains should be done to answer whether C5 is linked to a non-virulent complex from environment.

### Evolution of the pandemic group

The minimum spanning tree clearly showed that the pandemic clone (*trh*^-^, *tdh*^+ ^and GS-PCR^+^) was emerged from the old-O3:K6 clone (*trh*^+^, *tdh*^- ^and GS-PCR^-^) (Figure 2). One of the most interesting results was the identification of a phylogenetic intermediate (a non-pandemic O3:K6 strain S093) between the pandemic and old-O3:K6 clones (Figure 2).

Phylogenetically, strain S093 (*trh*^-^, *tdh*^- ^and GS-PCR^+^) was dramatically distant from the pandemic clone, but was closely related to the old-O3:K6 clone. Compared to the old-O3:K6 clone, a distinct feature of strain S093 was that it possessed the *toxRS*/new sequence (GS-PCR^+^). Similar pre-1996 O3:K6 strains (*trh*^-^, *tdh*^- ^and GS-PCR^+^) have been characterized previously [12]. All these strains were assigned to the proposed 'intermediate-O3:K6 clade'.

It was concluded herein that the acquisition of *toxRS*/new sequence led to the phylogenesis of the intermediate-O3:K6 clade from the old-O3:K6 clone, and that the post-1996 new O3:K6 stemmed from this intermediate clade after the acquisition of *tdh*, VpaI-5 and other unidentified genes (Figure 2).

The pandemic clone consisted of five serovars, O3:K6 (29 strains), O4:K68 (5), O1:K25 (3), O1:KUT (1) and O6:K18 (1). Slight genome plasticity was observed within the pandemic clone. According to the minimum spanning tree, the 39 pandemic strains were assigned into five sub-groups, SG1 to SG5 (Figure 2). Strains in a given sub-group gave almost identical genomic content. The 29 new-O3:K6 strains were included in SG1 to SG4. The remaining 10 strains of O4:K68, O1:K25, O1:KUT and O6:K18 fell into SG5. Date presented here provided the direct evidences to the notion that the post-1996 strains of O4:K68, O1:K25, O1:KUT and O6:K18 (called 'new-O3:K6 serovariants') were evolved from the new O3:K6.

Since strains in each of the five sub-groups (SG1 to SG5) gave almost identical genomic content, one strain was picked out from each sub-group. The M-GCH data (log_2 _ratios) of these representative strains were graphically shown in Additional file 4, which depicted the regions that present variably in the genome, particularly including genomic islands and O/K antigen genes.

**Figure 3:**
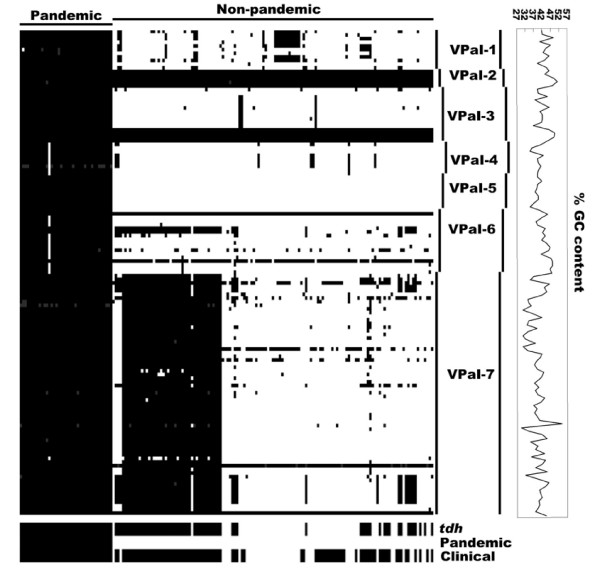
**Proposed evolutionary relatedness between old-O3:K6, intermediate-O3:K6, and new-O3:K6 and its serovariants**. Stepwise acquisition of genomic islands and the differentiation of O/K antigen genes promote the microevolution of the pandemic group (clone).

Taken all the above results together, a phylogenetic relatedness between 'old O3:K6', 'intermediate O3:K6', 'new O3:K6', and 'new-O3:K6 serovariants' were proposed (Figure 3). It was recently reported that, arising from old O3:K6, the pandemic clone acquired at least seven novel genomic regions including VPaI-1 to VpaI-7 plus a type VI secretion system (VP1386-1420) [20]. Data presented here (Figure 3) illustrated how the stepwise acquisition of genomic islands as well as the differentiation of O/K antigen genes promoted the microevolution of the pandemic clone, giving a proposed evolutionary history of the pandemic group.

### Variably-presented genes (VPGs)

#### GC contents

Of the 4021 genes surveyed, 871 were absent in at least one of the 174 strains tested. VPGs were often located continuously in the genome, generating various plasticity zones (Figure 1). As shown in Figure 1, the GC content of many genes in the plasticity zones, especially within the genomic islands, was lower than the average GC content of the entire genome.

Both variably- and universally-presented genes gave the same distribution of GC contents, i.e., the genes scattered from low to high GC contents [see Additional file 5]. However, the VPGs were over-presented and tailed towards low GC value. Thus, most genes with a GC content of less than 39% were variably presented. The 871 VPGs gave an average GC content of 44.1, whereas the average GC content of the remaining 3150 genes was 46.4. These results together indicated that many VPGs might be foreign origins through horizontal gene transfer.

#### Functional classification

The proportions of VPGs with respect to their functional category were shown in Additional file 6. Genes (1221 in total) responsible for house-keeping functions (small molecule or macromolecule metabolism) were relatively conserved; VPGs counted only 14% of them, especially including those involved in amino acid biosynthesis (7%), transcription (8%) and protein synthesis (8%). In contrast, 25% of the genes in the other six categories (cell envelope, transport and binding proteins, regulatory functions, cellular processes, mobile and extrachromosomal element functions, and unknown or unassigned function) were divergently distributed. It was surprising that 49% of the VPGs encoded unknown or unassigned functions. Genes in the category of mobile and extrachromosomal element function (phage- and transposon-related genes) exhibited the highest diversity; 96% of them were absent especially in non-clinical strains. These observations further supported the notion that VPGs were often imported from other species through horizontal gene transfer.

#### Large variably-presented gene clusters (VPCs)

While this manuscript was in preparation, a M-CGH study on 22 strains of *V. parahaemolyticus *was published by Izutsu et al [28]. The authors identified 13 large variably-presented gene clusters (VPC01 to VPC13) each containing more than 10 ORFs, all of which were confirmed by our results. Four additional large VPCs (VP1355-1368, VPA0074-0089, VPA0713-0732 and VPA1194-1210) were identified in the present work. However, the major difference between these two studies was that a much richer collection of isolates representing the global populations of *V. parahaemolyticus *were analyzed in this study for constructing a phylogenetic structure based on gene acquisition/loss, and to group the strains of distinct origins into multiple complexes, and to further propose the phylogeny of these complexes, especially including the evolutionary history of the pandemic group.

#### Genomic islands

**Figure 4:**
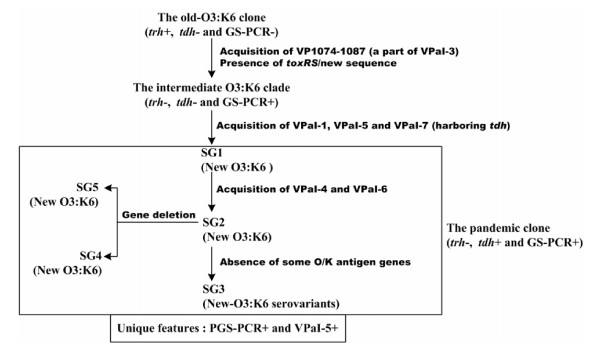
**Distribution of genomic islands**. Each column represents a strain, while each row stands for a different gene. The arrangement of strains is the same as that in Figure 1. For each strain, a black area indicates the presence of a gene, whereas white absence, and grey missing data. The GC content curve is shown on the right.

Based on the bioinformatics analysis, seven genomic islands (VPaI-1 to VPaI-7) were identified in the genome of strain RIMD2210633 [25]. Ranged in size from 10 kb to 81 kb, they are flanked by direct repeats. The first five are located on chromosome 1, while the later two on chromosome 2. Six of them (VPaI-1; VPaI-3 to VPaI-7) have a GC content (ranging from 38% to 43%) lower than the overall genome GC content of 45%; these six genomic islands were variably presented in the 174 strains tested (Figure 4). The remaining VPaI-2 with an average GC content (45%) equal to that of the whole genome was present in all the 174 strains tested (Figure 4). Absence of VPaI-2 was observed in *V. parahaemolyticus *as well [20]. Taken together, all the seven genomic islands were variably presented in *V. parahaemolyticus*, indicating the horizontal gene transfer of these elements among different linkages of *V. parahaemolyticus*.

A previous PCR screening analysis on 41 strains of *V. parahaemolyticus *showed that five (VPaI-1; VPaI-4 to VPaI-7) of the seven genomic islands were specific to the pandemic group [25]. Data presented here revealed that VPaI-1, VPaI-3, VPaI-4 and VPaI-6 were closely correlated to the pandemic strains (but not 100% correlation) (Figure 4). All the pandemic strains harbored VPaI-1 and VPaI-3, while VPaI-4 and VPaI-6 were absent from a single pandemic strain (S133) (Figure 4). All the above four were absent from most of the non-pandemic strains; although they were found in a few of non-pandemic strains, with only the partial components of each genomic island in most cases (Figure 4).

The 81 kb VPaI-7 encode a T3SS and two *tdh *genes. The M-CGH study by Izutsu et al [28] indicated that VPaI-7 was specific to KP^+^(*tdh*^+^) strains. Herein, 81 of the 105 *tdh*^+ ^strains harbored VPaI-7, and another *tdh*^- ^strain contained the gene cluster VPA1322-1369 (a part of VPaI-7) (Figure 4), suggesting that there was no 100% correlation between VPaI-7 and KP^+ ^(*tdh*^+^) phenotype.

The above results strongly argued that an analysis of a large collection of strains (so as to sufficiently represent the diversity of *V. parahaemolyticus*) was needed to make reasonable conclusions.

#### Pandemic clone-specific genes

Unlike some other bacterial pathogen, *V. parahaemolyticus *including its newly emerged pandemic clone has no unique plasmid. Identification of genomic regions specific to the pandemic clone will provide the signature DNA sequences for discrimination of pandemic strains from others. It is amazing that, except VPaI-5 (see above) no additional pandemic clone-specific genes (those present in all pandemic strains but not in all non-pandemic ones) were identified by M-CGH. VPaI-5(VP2900-2910) is a cluster of genes encoded hypothetical proteins with unknown function. At the present circumstance, the function or significance of VPaI-5 cannot be directly linked to the global outbreak of gastrointestinal infections caused by the pandemic group (clone).

#### O and K antigen genes

All genes in the genomic region VP0187-0238 were divergently distributed in the 174 strains. These genes are responsible for the biosynthesis of lipopolysaccharides and capsular polysaccharides that determine major O and K antigens of *V. parahaemolyticus*. Similar results were found in Izutsu et al' M-CGH study [28]. The above results indicated deletion or horizontal transfer of relevant genes accounted for the diversification of O and K antigens of this bacterium.

### Concluding remarks

The previous PFGE, AP-PCR and MLST studies showed the significant genetic variability within the populations of *V. parahaemolyticus*, but the pandemic group members were highly conserved genetically to form a clonal complex [5-9]. Although the wide use of traditional typing systems for the epidemiological analysis of *V. parahaemolyticus*, a clear disagreement has been observed between O:K serovars and FP/STs. Strains of a single serovar (e.g. O3:K6) often gave distinct FP/STs, whereas a number of serovars among the pandemic group gave similar or undistinguishable FP/STs. It is apparent that the serotyping scheme is not reliable for characterizing the epidemiological spread of *V. parahaemolyticus*. In addition, both serotyping and traditional molecular typing methods are limited in accurately tracking genetic differences and phylogenetic relatedness of strains. In the present work, a comprehensive overview of genomic contents in a large collection of global isolates from the M-CGH data enabled us to construct a phylogenetic structure of *V. parahaemolyticus*, which had not been previously detected with traditional typing systems. This would enhance the understanding of molecular epidemiology and evolution of this pathogen. Overall, the considerable gene acquisition/loss promoted the genetic diversification of *V. parahaemolyticus *strains to form distinct clonal or semi-clonal complexes. In particular, we identified two different clonal complexes 'old-O3:K6 clone' and 'pandemic clone'. The pandemic clone included all the 39 pandemic group members tested, which confirmed the previous notion [10-12]. It is the first report of the old-O3:K6 clone consisting of 12 pre-1996 old O3:K6 strains tested herein.

A major conclusion of this study was the depiction of an evolutionary history of the pandemic group (clone). Strains of new-O3:K6 and its serovariants (post-1996 O4:K68, O1:K25, O1:KUT and O6:K18) constituted the pandemic group. New-O3:K6 was emerged from the old-O3:K6 clone by the stepwise acquisition of genomic islands. A small group of O3:K6 strains (named as the intermediate-O3:K6 clade) served as the phylogenetic intermediate between new-O3:K6 and old-O3:K6. The differentiation of O/K antigen genes promoted the derivation of new-O3:K6 serovariants from new-O3:K6.

## Materials and methods

### Bacterial strains

One hundred and seventy four strains of *V. parahaemolyticus *were used in the M-CGH assay [see Additional file 1]. Two of them, S072 and S004, were used as reference strains in microarray analysis. Strain S072 is a post-1996 pandemic O3:K6 isolate, while the later one is a pre-1996 O3:K6 strain. Bacteria were grown in the LB-2% NaCl agar at 37°C, and the extraction of genomic DNA was performed by the classical phenol/chloroform method.

### Construction of DNA microarray

Gene-specific primer pair was designed to amplify the almost whole-length of each annotated gene of strain RIMD2210633. A total of 4660 genes representing about 96% of *V. parahaemolyticus *genome were amplified successfully, using the genomic DNA of strain S072 or S004 as template. The purified PCR products were spotted in duplicate on the CSS-1000 silylated glass slides (CEL) by using a SpotArray72 Microarray Printing System (Perkin Elmer Life Sciences) with 32 Telechem SMP3 Stealth Pins (4 × 8 Layout) to construct the DNA microarrays.

### DNA labeling and microarray hybridization

The genomic DNA mixture of S072 and S004 with equal quantity was used as 'Reference DNA'. Genomic DNA from each of the *V. parahaemolyticus *isolates studied was referred to as 'Test DNA'. Cy3- or Cy5- labeled probes were generated by priming of the Reference or Test DNA with random hexamers and extension with Klenow [27]. The labeled Reference and Test DNA were combined to hybridize with the microarrays by the dual-fluorescence hybridization method [27]. All hybridizations were performed in duplicate. Pairwise comparisons were made for each strain using dye swaps to avoid labeling bias.

### Microarray data mining

The hybridized slides were scanned by using a GenePix Personal 4100A Microarray Scanner (Axon Instruments). The scanning images were processed and the data were further analyzed by using GenePix Pro 5.0 software (Axon Instruments) combined with Microsoft Excel software. Spots with signal intensity (median) in the channel of Reference DNA less than two folds of local background intensity (median) were rejected from further analysis. Spots with bad data because of slide abnormalities were discarded as well. Data normalization was performed on the remaining spots by total intensity normalization methods. A ratio of intensity (Test DNA normalized intensity/Reference DNA normalized intensity) was recorded for each spot and then was converted to log_2_. Genes with fewer than three data points were considered unreliable, and were accordingly removed. The averaged log_2 _ratio for each remaining gene on the two replicate slides was ultimately calculated. If 20% of the strains had a gene with missing data, the gene was removed. A total of 4021 genes were included in the final dataset. A log_2 _value equal to or lower than -1 was taken as defining the absence of a gene in given strain.

### Clustering and phylogenetic analysis

The final absent (0) or present (1) call was assigned to each gene for each strain in the M-CGH data, and analyzed by BioNumerics Version 5.01 (Applied Maths). Clustering was carried out subsequently by the unweighted-pair group method using average linkages (UPGMA), to calculate a similarity matrix. A minimum spanning tree was built based on the similarity matrix. The clustered microarray data were displayed by the TreeView tool [29].

### PCR analysis

Bacterial genomic DNAs to be tested were arrayed in 96-well PCR plates. Each gene-specific primer pair [see Additional file 2] was pre-tested with the Reference DNA as template, to ensure the successful amplification. A volume of 25 μl PCR mixture contained 50 mM KCl, 10 mM Tris-HCl (pH8.0), 2.5 mM MgCl_2_, 0.001% gelatin, 0.1% BSA, 100 μM of each dATP, dCTP, dGTP and dTTP, 0.1 μM of each primer, 1 unit of *Taq *DNA polymerase (MBI), and 10 ng of template DNA. The parameters for amplification were as follows: 95°C for 3 min; 30 cycles of 94°C for 30 s, an appropriate annealing temperature for 30 s, and 72°C for 1 min; and a final extension step of 72°C for 5 min. PCR products were analyzed by 1.2% agarose gel electrophoresis with ethidium bromide staining.

## Authors' contributions

RY, DZ and XL conceived the study and designed the experiments. The manuscript was written by DZ, and revised by RY and HW. All the authors performed the experiments, and read and approved the final manuscript.

## Supplementary Material

Additional file 1 **The 174 strains of *Vibrio parahaemolyticus *used in this study.** Click here for file

Additional file 2 **Primers used in PCR analysis.** Click here for file

Additional file 3 **Distribution of *tdh *and *trh *genes in the 174 strains.** Click here for file

Additional file 4 **The M-GCH data of representative strains, S008, S093, S133, S068, S087, S080, and S082.** Click here for file

Additional file 5 **Distribution of variably- and universally-present genes according to their GC composition.** Click here for file

Additional file 6 **Distribution of variably-present genes according to their functional category.** Click here for file
