# Supplementary material for: Genome plasticity of Vibrio parahaemolyticus: microevolution of the 'pandemic group'
Source: BMC Genomics. 2008 Nov 28;9:570. doi: 10.1186/1471-2164-9-570 (PMC2612023; doi:10.1186/1471-2164-9-570)
Supplement: Additional file 1 — The 174 strains of Vibrio parahaemolyticus used in this study. [file 1471-2164-9-570-S1.doc]

**Additional file 1. Strains of *Vibrio parahaemolyticus* used in this study**

| **ID** | **Strain** | **Year** | **Serovar** | **Region** | **Complex** | **Clinical** | **Pandemic** | **VPA1322-1369** | ***tdh*** | ***trh*** | **VPaI-5** |
| --- | --- | --- | --- | --- | --- | --- | --- | --- | --- | --- | --- |
| S001 | 1469 | Unkown | ND | Thailand | C5 | 1 | 0 | 0 | 0 | 0 | 0 |
| S002 | 1293 | 1999 | O2:K3 | Taiwan, China | C4 | 1 | 0 | 1 | 1 | 0 | 0 |
| S003 | 1185 | Unknown | O3:K6 | Hong Kong, China | C2 | 1 | 0 | 0 | 0 | 1 | 0 |
| S004 | 1240 | 1985 | O3:K6 | Maldives | C2 | 1 | 0 | 0 | 0 | 1 | 0 |
| S005 | 1186 | Unknown | O3:K6 | Thailand | C2 | 1 | 0 | 0 | 0 | 1 | 0 |
| S006 | 1175 | 1983 | O3:K6 | Singapore | C2 | 1 | 0 | 0 | 0 | 1 | 0 |
| S007 | 1177 | 1985 | O3:K6 | Maldives | C2 | 1 | 0 | 0 | 0 | 1 | 0 |
| S008 | 1181 | 1987 | O3:K6 | Thailand | C2 | 1 | 0 | 0 | 0 | 1 | 0 |
| S009 | 1182 | 1987 | O3:K6 | Thailand | C2 | 1 | 0 | 0 | 0 | 1 | 0 |
| S010 | 1449 | 1984 | O3:K6 | Japan | C2 | 1 | 0 | 0 | 0 | 1 | 0 |
| S011 | 1477 | 1990 | O3:K6 | Thailand | C2 | 1 | 0 | 0 | 0 | 1 | 0 |
| S012 | 1475 | 1990 | O3:K6 | Thailand | C2 | 1 | 0 | 0 | 0 | 1 | 0 |
| S013 | 1304 | 1996 | O3:K6 | Taiwan, China | C2 | 1 | 0 | 0 | 0 | 1 | 0 |
| S014 | 133 | 1992 | ND | Taiwan, China | C4 | 1 | 0 | 1 | 1 | 0 | 0 |
| S015 | 134 | 1992 | ND | Taiwan, China | C4 | 1 | 0 | 1 | 1 | 0 | 0 |
| S016 | 166 | 1992 | O3:K29 | Taiwan, China | C4 | 1 | 0 | 1 | 1 | 0 | 0 |
| S017 | 141 | 1992 | ND | Taiwan, China | C4 | 1 | 0 | 1 | 1 | 0 | 0 |
| S018 | 304 | 1993 | O3:K29 | Taiwan, China | C4 | 1 | 0 | 1 | 1 | 0 | 0 |
| S019 | 1400 | 1998 | O3:K36 | USA | C1 | 1 | 0 | 0 | 1 | 1 | 0 |
| S020 | 634 | 1994 | O5:K15 | Taiwan, China | C4 | 1 | 0 | 1 | 1 | 0 | 0 |
| S021 | 1373 | 1999 | O5:K15 | India | C4 | 1 | 0 | 1 | 1 | 0 | 0 |
| S022 | 1442 | 1984 | O5:K15 | Japan | C4 | 1 | 0 | 1 | 1 | 0 | 0 |
| S023 | 643 | 1994 | O3:K5 | Taiwan, China | C4 | 1 | 0 | 1 | 1 | 1 | 0 |
| S024 | 1330 | 1998 | O4:K10 | Taiwan, China | C4 | 1 | 0 | 1 | 1 | 1 | 0 |
| S025 | 145 | 1992 | ND | Taiwan, China | C4 | 1 | 0 | 1 | 1 | 0 | 0 |
| S026 | 1465 | Unkown | ND | Thailand | C4 | 1 | 0 | 1 | 1 | 0 | 0 |
| S027 | 1410 | Unkown | ND | USA | C4 | 1 | 0 | 1 | 1 | 0 | 0 |
| S028 | 1466 | Unkown | ND | Thailand | C4 | 1 | 0 | 1 | 1 | 0 | 0 |
| S029 | 1419 | 1981 | ND | Spain | C5 | 0 | 0 | 0 | 0 | 0 | 0 |
| S030 | 1379 | 1999 | O4:K8 | India | C4 | 1 | 0 | 1 | 1 | 0 | 0 |
| S031 | 1441 | 1984 | O4:K13 | Japan | C4 | 1 | 0 | 1 | 1 | 0 | 0 |
| S032 | 1261 | 1998 | O1:K56 | Phillipine | C4 | 1 | 0 | 1 | 1 | 0 | 0 |
| S033 | 672 | 1994 | O5:K60 | Taiwan, China | C4 | 1 | 0 | 1 | 1 | 0 | 0 |
| S034 | 1381 | 1999 | O5:KUT | India | C4 | 1 | 0 | 1 | 1 | 0 | 0 |
| S035 | 1445 | 1984 | O4:K53 | Japan | C4 | 1 | 0 | 1 | 1 | 0 | 0 |
| S036 | 1461 | Unknown | ND | Thailand | C4 | 1 | 0 | 1 | 1 | 0 | 0 |
| S037 | 1389 | 1994 | O4:K12 | USA | C1 | 1 | 0 | 0 | 1 | 1 | 0 |
| S038 | 1390 | 1982 | O4:K12 | USA | C1 | 0 | 0 | 0 | 1 | 1 | 0 |
| S039 | 1036 | Unkown | ND | Taiwan, China | C5 | 1 | 0 | 0 | 0 | 0 | 0 |
| S040 | 1471 | 1990 | O1:K25 | Thailand | C5 | 1 | 0 | 0 | 0 | 1 | 0 |
| S041 | 548 | 1993 | O4:K63 | Taiwan, China | C4 | 1 | 0 | 1 | 1 | 0 | 0 |
| S042 | 1436 | 1984 | O3:K6 | Japan | C4 | 1 | 0 | 1 | 1 | 0 | 0 |
| S043 | 1478 | Unknown | Unknown | Thailand | C1 | 1 | 0 | 0 | 0 | 1 | 0 |
| S044 | 1481 | 1990 | O1:K69 | Thailand | C1 | 1 | 0 | 0 | 1 | 1 | 0 |
| S045 | 1482 | 1990 | O1:K69 | Thailand | C1 | 1 | 0 | 0 | 1 | 1 | 0 |
| S046 | 1420 | 1982 | ND | Spain | C1 | 0 | 0 | 0 | 0 | 0 | 0 |
| S047 | 1418 | 1981 | ND | Spain | C5 | 0 | 0 | 0 | 0 | 0 | 0 |
| S048 | 1408 | 1997 | O11:KUT | USA | C1 | 0 | 0 | 0 | 0 | 0 | 0 |
| S049 | 1438 | 1984 | O4:K8 | Japan | C4 | 1 | 0 | 1 | 1 | 0 | 0 |
| S050 | 1426 | Unknown | ND | USA | C5 | 0 | 0 | 0 | 0 | 0 | 0 |
| S051 | 1427 | Unknown | ND | USA | C5 | 0 | 0 | 0 | 0 | 0 | 0 |
| S052 | 1417 | 1975 | ND | Spain | Unassigned | 0 | 0 | 0 | 0 | 0 | 0 |
| S053 | 1487 | 1990 | O1:K69 | Thailand | C1 | 1 | 0 | 0 | 1 | 1 | 0 |
| S054 | 1467 | Unknown | ND | Thailand | C4 | 1 | 0 | 1 | 1 | 0 | 0 |
| S055 | 720 | 1994 | O1:K41 | Taiwan, China | C4 | 1 | 0 | 1 | 1 | 0 | 0 |
| S056 | 668 | 1994 | O1:K56 | Taiwan, China | C4 | 1 | 0 | 1 | 1 | 0 | 0 |
| S057 | 718 | 1994 | O1:K41 | Taiwan, China | C4 | 1 | 0 | 1 | 1 | 0 | 0 |
| S058 | 1395 | 1970 | O4:K12 | Japan | C1 | 1 | 0 | 0 | 1 | 1 | 0 |
| S059 | 1394 | 1997 | O4:K29 | USA | C1 | 0 | 0 | 0 | 1 | 1 | 0 |
| S060 | 196 | 1992 | ND | Taiwan, China | C1 | 1 | 0 | 0 | 0 | 0 | 0 |
| S061 | 1262 | 1998 | O3:K6 | Singapore | C3 | 1 | 1 | 1 | 1 | 0 | 1 |
| S062 | 1263 | 1998 | O6:K18 | Singapore | C3 | 1 | 1 | 1 | 1 | 0 | 1 |
| S063 | 1346 | 1998 | O1:K25 | Taiwan, China | C3 | 1 | 1 | 1 | 1 | 0 | 1 |
| S064 | 1347 | 1998 | O3:K6 | Taiwan, China | C3 | 1 | 1 | 1 | 1 | 0 | 1 |
| S065 | 1267 | 1998 | O1:K25 | Taiwan, China | C3 | 1 | 1 | 1 | 1 | 0 | 1 |
| S066 | 1152 | 1997 | O3:K6 | Taiwan, China | C3 | 1 | 1 | 1 | 1 | 0 | 1 |
| S067 | 1153 | 1997 | O3:K6 | Taiwan, China | C3 | 1 | 1 | 1 | 1 | 0 | 1 |
| S068 | 1155 | 1997 | O3:K6 | Taiwan, China | C3 | 1 | 1 | 1 | 1 | 0 | 1 |
| S069 | 1463 | Unknown | O3:K6 | Thailand | C3 | 1 | 1 | 1 | 1 | 0 | 1 |
| S070 | 1468 | Unknown | O3:K6 | Thailand | C3 | 1 | 1 | 1 | 1 | 0 | 1 |
| S071 | 1247 | 1998 | O1:KUT | Bangladesh | C3 | 1 | 1 | 1 | 1 | 0 | 1 |
| S072 | 1248 | 1998 | O3:K6 | Bangladesh | C3 | 1 | 1 | 1 | 1 | 0 | 1 |
| S073 | 1023 | 1997 | O3:K6 | Thailand | C4 | 1 | 0 | 1 | 1 | 0 | 0 |
| S074 | 1139 | 1997 | O3:K6 | Taiwan, China | C3 | 1 | 1 | 1 | 1 | 0 | 1 |
| S075 | 1227 | 1999 | O3:K6 | Taiwan, China | C3 | 1 | 1 | 1 | 1 | 0 | 1 |
| S076 | 1228 | 1999 | O3:K6 | Taiwan, China | C3 | 1 | 1 | 1 | 1 | 0 | 1 |
| S077 | 1229 | 1999 | O3:K6 | Taiwan, China | C3 | 1 | 1 | 1 | 1 | 0 | 1 |
| S078 | 1230 | 1999 | O3:K6 | Taiwan, China | C3 | 1 | 1 | 1 | 1 | 0 | 1 |
| S079 | 1221 | Unknown | O3:K6 | Indonesia | C3 | 1 | 1 | 1 | 1 | 0 | 1 |
| S080 | 1084 | 1997 | O3:K6 | Taiwan, China | C3 | 1 | 1 | 1 | 1 | 0 | 1 |
| S081 | 1203 | Unknown | O3:K6 | Korea | C3 | 1 | 1 | 1 | 1 | 0 | 1 |
| S082 | 1458 | Unknown | O3:K6 | Thailand | C3 | 1 | 1 | 1 | 1 | 0 | 1 |
| S083 | 1249 | 1998 | O3:K6 | Japan | C3 | 1 | 1 | 1 | 1 | 0 | 1 |
| S084 | 1172 | 1996 | O3:K6 | Singapore | C3 | 1 | 1 | 1 | 1 | 0 | 1 |
| S085 | 1173 | 1996 | O3:K6 | Thailand | C3 | 1 | 1 | 1 | 1 | 0 | 1 |
| S086 | 1259 | 1999 | O1:K25 | Thailand | C3 | 1 | 1 | 1 | 1 | 0 | 1 |
| S087 | 1264 | 1998 | O4:K68 | Singapore | C3 | 1 | 1 | 1 | 1 | 0 | 1 |
| S088 | 1264 | 1998 | O4:K68 | Singapore | C3 | 1 | 1 | 1 | 1 | 0 | 1 |
| S089 | 1265 | 1998 | O4:K68 | Singapore | C3 | 1 | 1 | 1 | 1 | 0 | 1 |
| S090 | 1362 | 1999 | O4:K68 | Taiwan, China | C3 | 1 | 1 | 1 | 1 | 0 | 1 |
| S091 | 1377 | 1999 | O4:K68 | India | C3 | 1 | 1 | 1 | 1 | 0 | 1 |
| S092 | 1291 | 1996 | O3:K6 | Taiwan, China | C3 | 1 | 1 | 1 | 1 | 0 | 1 |
| S093 | 1252 | 1998 | O3:K6 | Japan | Unassigned | 1 | 0 | 0 | 0 | 0 | 0 |
| S094 | 1456 | 1996 | O3:K6 | Thailand | C3 | 1 | 1 | 1 | 1 | 0 | 1 |
| S095 | 1301 | 1996 | O3:K6 | Taiwan, China | C2 | 1 | 0 | 0 | 0 | 1 | 0 |
| S096 | 1283 | 1999 | O3:K6 | Korea | C4 | 1 | 0 | 1 | 0 | 0 | 0 |
| S097 | 135 | 1992 | ND | Taiwan, China | C1 | 1 | 0 | 0 | 0 | 0 | 0 |
| S098 | 1401 | 1997 | O3:K48 | USA | C1 | 0 | 0 | 0 | 0 | 0 | 0 |
| S099 | 1406 | 1991 | O8:K? | USA | C1 | 1 | 0 | 0 | 1 | 1 | 0 |
| S100 | 1392 | 1990 | O4:K49 | USA | Unassigned | 0 | 0 | 0 | 1 | 1 | 0 |
| S101 | 1393 | 1990 | O4:K63 | USA | Unassigned | 0 | 0 | 0 | 1 | 1 | 0 |
| S102 | 1396 | 1991 | O1:K56 | USA | Unassigned | 1 | 0 | 0 | 1 | 1 | 0 |
| S103 | 1386 | 1997 | O4:K12 | USA | Unassigned | 1 | 0 | 0 | 1 | 1 | 0 |
| S104 | 1405 | 1997 | O6:K18 | USA | C1 | 1 | 0 | 0 | 1 | 1 | 0 |
| S105 | 1439 | 1984 | O4:K9 | Japan | C1 | 1 | 0 | 0 | 0 | 1 | 0 |
| S106 | 1485 | 1990 | O5:KUT | Thailand | C5 | 1 | 0 | 0 | 1 | 1 | 0 |
| S107 | 1457 | Unknown | ND | Thailand | C4 | 1 | 0 | 1 | 1 | 0 | 0 |
| S108 | 1473 | 1990 | O1:KUT | Thailand | C5 | 1 | 0 | 0 | 1 | 1 | 0 |
| S109 | 527 | 1993 | O4:K10 | Taiwan, China | C4 | 1 | 0 | 1 | 1 | 0 | 0 |
| S110 | 1380 | 1999 | OUT:K1 | India | C1 | 1 | 0 | 0 | 1 | 1 | 0 |
| S111 | 1479 | 1990 | O1:K1 | Thailand | C1 | 1 | 0 | 0 | 1 | 1 | 0 |
| S112 | 1416 | 1951 | ND | Japan | C1 | 1 | 0 | 0 | 0 | 1 | 0 |
| S113 | 1483 | 1990 | O1:K1 | India | C1 | 1 | 0 | 0 | 1 | 1 | 0 |
| S114 | 1484 | 1998 | O2:K28 | India | C1 | 1 | 0 | 0 | 1 | 1 | 0 |
| S115 | 1489 | 1991 | O1:K69 | Thailand | C1 | 1 | 0 | 0 | 0 | 1 | 0 |
| S116 | 1464 | Unknown | ND | Thailand | C1 | 1 | 0 | 0 | 1 | 0 | 0 |
| S117 | 1452 | 1984 | KUT | Japan | C1 | 1 | 0 | 0 | 1 | 1 | 0 |
| S118 | 1437 | 1984 | O4:K8 | Japan | C4 | 1 | 0 | 1 | 1 | 0 | 0 |
| S119 | 1364 | 1999 | O4:K11 | Taiwan, China | C4 | 1 | 0 | 1 | 1 | 0 | 0 |
| S120 | 1480 | 1990 | O10:KUT | Thailand | C5 | 1 | 0 | 0 | 1 | 1 | 0 |
| S121 | 1496 | Unknown | ND | Thailand | C4 | 1 | 0 | 1 | 1 | 1 | 0 |
| S122 | 1376 | 1999 | O1:K38 | India | C4 | 1 | 0 | 1 | 1 | 0 | 0 |
| S123 | 1433 | 1984 | O1:K64 | Japan | C4 | 1 | 0 | 1 | 1 | 0 | 0 |
| S124 | 198 | 1992 | O3:K57 | Taiwan, China | C4 | 1 | 0 | 1 | 1 | 0 | 0 |
| S125 | 1391 | 1997 | O4:K13 | USA | C1 | 0 | 0 | 0 | 0 | 0 | 0 |
| S126 | 1188 | Unknown | O3:K6 | Taiwan, China | C3 | 1 | 1 | 1 | 1 | 0 | 1 |
| S127 | 169 | 1992 | ND | Taiwan, China | C5 | 1 | 0 | 0 | 0 | 0 | 0 |
| S128 | 1374 | 1999 | O8:KUT | India | C1 | 1 | 0 | 0 | 0 | 0 | 0 |
| S129 | 1446 | 1984 | O5:K60 | Japan | C1 | 1 | 0 | 0 | 1 | 1 | 0 |
| S130 | Shen | 2003 | ND | China | C4 | 1 | 0 | 1 | 1 | 0 | 0 |
| S131 | Qin | 2003 | O3:K6 | China | C3 | 1 | 1 | 1 | 1 | 0 | 1 |
| S132 | ICDC-VP32 | 2005 | ND | Niaoning, China | C4 | 1 | 0 | 1 | 1 | 0 | 0 |
| S133 | ICDC-VP53 | 2005 | O3:K6 | Niaoning, China | C3 | 1 | 1 | 1 | 1 | 0 | 1 |
| S134 | ICDC-VP75 | 2005 | ND | Niaoning, China | C4 | 1 | 0 | 1 | 1 | 0 | 0 |
| S135 | ICDC-VP77 | 2003 | O3:K6 | Guangxi, China | C3 | 1 | 1 | 1 | 1 | 0 | 1 |
| S136 | ICDC-VP87 | 2004 | O3:K6 | Guangxi, China | C3 | 1 | 1 | 1 | 1 | 0 | 1 |
| S137 | ICDC-VP88 | 2005 | O3:K6 | Guangxi, China | C3 | 1 | 1 | 1 | 1 | 0 | 1 |
| S138 | ICDC-VP133 | 2007 | O3:K6 | Hebei, China | C3 | 1 | 1 | 1 | 1 | 0 | 1 |
| S139 | 06VP240 | 2006 | ND | Inner Mongolia, China | C1 | 0 | 0 | 0 | 0 | 0 | 0 |
| S140 | 06VP271 | 2006 | ND | Shanghai, China | C1 | 0 | 0 | 0 | 0 | 0 | 0 |
| S141 | 06VP283 | 2006 | ND | Shanghai, China | C5 | 0 | 0 | 0 | 0 | 0 | 0 |
| S142 | 06VP297 | 2006 | ND | Shanghai, China | C5 | 0 | 0 | 0 | 0 | 0 | 0 |
| S143 | 06VP373 | 2006 | ND | Shandong, China | C5 | 0 | 0 | 0 | 0 | 0 | 0 |
| S144 | 06VP383 | 2006 | ND | Shandong, China | C5 | 0 | 0 | 0 | 0 | 0 | 0 |
| S145 | 06VP241 | 2006 | ND | Inner Mongolia, China | C1 | 0 | 0 | 0 | 0 | 0 | 0 |
| S146 | 06VP248 | 2006 | ND | Inner Mongolia, China | C5 | 0 | 0 | 0 | 0 | 0 | 0 |
| S147 | 06VP256 | 2006 | ND | Inner Mongolia, China | C5 | 0 | 0 | 0 | 0 | 0 | 0 |
| S148 | 06VP257 | 2006 | ND | Inner Mongolia, China | C5 | 0 | 0 | 0 | 0 | 0 | 0 |
| S149 | 06VP288 | 2006 | ND | Shanghai, China | C5 | 0 | 0 | 0 | 0 | 0 | 0 |
| S150 | 06VP368 | 2006 | ND | Shandong, China | C5 | 0 | 0 | 0 | 0 | 0 | 0 |
| S151 | 06VP369 | 2006 | ND | Shandong, China | C5 | 0 | 0 | 0 | 0 | 0 | 0 |
| S152 | 06VP370 | 2006 | ND | Shandong, China | C1 | 0 | 0 | 0 | 0 | 0 | 0 |
| S153 | 06VP371 | 2006 | ND | Shandong, China | C5 | 0 | 0 | 0 | 0 | 0 | 0 |
| S154 | 06VP374 | 2006 | ND | Shandong, China | C5 | 0 | 0 | 0 | 0 | 0 | 0 |
| S155 | 06VP377 | 2006 | ND | Shandong, China | C5 | 0 | 0 | 0 | 0 | 0 | 0 |
| S156 | 06VP379 | 2006 | ND | Shandong, China | C5 | 0 | 0 | 0 | 0 | 0 | 0 |
| S157 | 06VP382 | 2006 | ND | Shandong, China | Unassigned | 0 | 0 | 0 | 0 | 0 | 0 |
| S158 | 06VP384 | 2006 | ND | Shandong, China | C5 | 0 | 0 | 0 | 0 | 0 | 0 |
| S159 | 06VP385 | 2006 | ND | Shandong, China | Unassigned | 0 | 0 | 0 | 0 | 0 | 0 |
| S160 | 06VP386 | 2006 | ND | Shandong, China | Unassigned | 0 | 0 | 0 | 0 | 0 | 0 |
| S161 | 06VP387 | 2006 | ND | Shandong, China | C5 | 0 | 0 | 0 | 0 | 0 | 0 |
| S162 | ICDC-VP29 | 2007 | ND | Thailand | C5 | 0 | 0 | 0 | 0 | 0 | 0 |
| S163 | ICDC-VP30 | 2007 | ND | Malaysia | C1 | 0 | 0 | 0 | 0 | 0 | 0 |
| S164 | ICDC-VP31 | 2007 | ND | USA | C1 | 0 | 0 | 0 | 1 | 0 | 0 |
| S165 | ICDC-VP90 | 2007 | ND | Shanghai, China | C5 | 0 | 0 | 0 | 0 | 0 | 0 |
| S166 | ICDC-VP92 | 2007 | ND | Shanghai, China | C5 | 0 | 0 | 0 | 0 | 0 | 0 |
| S167 | ICDC-VP93 | 2007 | ND | Shanghai, China | C1 | 0 | 0 | 0 | 0 | 0 | 0 |
| S168 | ICDC-VP94 | 2007 | ND | Shanghai, China | C5 | 0 | 0 | 0 | 0 | 0 | 0 |
| S169 | ICDC-VP95 | 2007 | ND | Shanghai, China | C1 | 0 | 0 | 0 | 0 | 0 | 0 |
| S170 | ICDC-VP101 | 2007 | ND | Shanghai, China | C5 | 0 | 0 | 0 | 0 | 0 | 0 |
| S171 | ICDC-VP106 | 2007 | ND | Shanghai, China | C5 | 0 | 0 | 0 | 0 | 0 | 0 |
| S172 | ICDC-VP107 | 2007 | ND | Shanghai, China | C5 | 0 | 0 | 0 | 0 | 0 | 0 |
| S173 | ICDC-VP108 | 2007 | ND | Shanghai, China | C5 | 0 | 0 | 0 | 0 | 0 | 0 |
| S174 | ICDC-VP110 | 2007 | ND | Shanghai, China | C1 | 0 | 0 | 0 | 0 | 0 | 0 |

42
