# Supplementary material for: Genome plasticity of Vibrio parahaemolyticus: microevolution of the 'pandemic group'
Source: BMC Genomics. 2008 Nov 28;9:570. doi: 10.1186/1471-2164-9-570 (PMC2612023; doi:10.1186/1471-2164-9-570)
Supplement: Additional file 2 — Primers used in PCR analysis. [file 1471-2164-9-570-S2.doc]

**Additional file 2. Primers used in PCR analysis**

| **Marker** | **Primer name** | **Size of amplicon** | **Annealing temperature** | **Primer sequence** | **Reference** |
| --- | --- | --- | --- | --- | --- |
| **For detection of various DNA markers** | | | | | |
| *toxR* | toxR367F | 367 | 60 | GACTTCTGACGCAATCGTTG | [1] |
|  | toxR367R |  |  | ATACGAGTGGTTGCTGTCATG |  |
| *gyrB* | VP-1 | 285 | 58 | CGGCGTGGGTGTTTCGGTAGT | [2] |
|  | VP-2r |  |  | TCCGCTTCGCGCTCATCAATA |  |
| vpm | VPM1 | 675 | 60 | CAGCTACCGAAACAGACGCTA | [3] |
|  | VPM2 |  |  | TCCTATCGAGGACTCTCTCAAC |  |
| *tl* | tl450F | 450 | 58 | AAAGCGGATTATGCAGAAGCACTG | [4, 5] |
|  | tl450R |  |  | GCTACTTTCTAGCATTTTCTCTGC |  |
| *tdh* | tdh251F | 251 | 55 | GGTACTAAATGGCTGACATC | [6] |
|  | tdh251R |  |  | CCACTACCACTCTCATATGC |  |
|  | tdh269F | 269 | 58 | GTAGAGGTCTCTGACTTTTGGAC | [7] |
|  | tdh269R |  |  | TGGAATAGAACCTTCATCTTCACC |  |
| *trh* | trh250F | 250 | 55 | GGCTCAAAATGGTTAAGCG | [6] |
|  | trh250R |  |  | CATTTCCGCTCTCATATGC |  |
|  | trh487F | 487 | 58 | TTGCTTTCAGTTTGCTATTGG | This study |
|  | trh487R |  |  | CCATTGCCGCTCTCATATGC |  |
| GS-PCR | GS-VP.1 | 651 | 45 | TAATGAGGTAGAAACA | [8] |
|  | GS-VP.2 |  |  | ACGTAACGGGCCTACA |  |
| PGS-PCR | PGS-1 | 235 | 60 | TTCGTTTCGCGCCACAACT | [9] |
|  | PGS-2 |  |  | TGCGGTGATTATTCGCGTCT |  |
| ORF8 | 8S |  | 57 | GTTCGCATACAGTTGAGG | [10] |
|  | 8A |  |  | AAGTACAGCAGGAGTGAG |  |
| HU-α | HU-α-F | 474 | 58 | CGATAACCTATGAGAAGGGAAACC | [11] |
|  | HU-α-R |  |  | CTAGTAAGGAAGAATTGATTGTCAAATAATG |  |
| **For validation of microarray relsults of VPA1322-1369** | | | | | |
| VPA1323 | VPA1323F | 218 | 54 | AAGGTTAGGAGCCTGTTGAG | This study |
|  | VPA1323R |  |  | AAGATTTTGTAGCCTACCCC |  |
| VPA1327 | VPA1327F | 550 | 56 | CTAGTGCTCGTGCGATTAAC | This study |
|  | VPA1327R |  |  | TCAAGTAAGGCAGGCACATG |  |
| VPA1333 | VPA1333F | 419 | 56 | GATTGCACCTGAGCTTAAC | This study |
|  | VPA1333R |  |  | CACAACCACGTACTCTTCG |  |
| VPA1353 | VPA1353F | 700 | 56 | CCGGGTGCAGTAGTCAAATC | This study |
|  | VPA1353R |  |  | TGCTGAGCAGTCGATAAACC |  |
| **For validation of microarray relsults of VP2900-2910 (VPaI-5）** | | | | | |
| VP2900 | PVP2900F | 300 | 54 | GGCAAAGACTCGGAAGGTGAG | This study |
|  | PVP2900R |  |  | AGCAGGCAGGTGTTCTTCTAG |  |
| VP2901 | VP2901F | 923 | 54 | AGTCCTCCCGTCGAAAGC | This study |
|  | VP2901R |  |  | ACCCACGACAGGAATCCC |  |
| VP2902 | VP2902F | 907 | 54 | AGTCGCAGGAATAAGAGACG | This study |
|  | VP2902R |  |  | GTTCAAGAGCATCGCAATCC |  |
| VP2903 | VP2903F | 1,135 | 54 | GGGGCTAGGCAAGACCG | This study |
|  | VP2903R |  |  | GGCTTCACCCTCCCACC |  |
| VP2905 | VP2905F | 2,000 | 54 | TACTGGCTGTCGAACTCC | This study |
|  | VP2905R |  |  | TGTTGCAGGCTGGTCTG |  |
| VP2907 | VP2907F | 360 | 54 | AGCATTTTAAGCCAGTTCG | This study |
|  | VP2907R |  |  | AGCTCTCAAAAGAGGAGAC |  |
| VP2908 | PVP2908F | 300 | 54 | AGCTAAGGTGATTGGCAAGGC | This study |
|  | PVP2908R |  |  | TCTCGTTCAGCAGTAACTCGC |  |
| VP2909 | VP2909F | 980 | 54 | TGGGGCCATTTACTCGTG | This study |
|  | VP2909R |  |  | CGATGTAGGCGAGCTTGC |  |
| VP2910 | PVP2910F | 300 | 54 | TTGGGACGCGAAATTGAACTC | This study |
|  | PVP2910R |  |  | CTTCAACTTCCGGCTCAATGG |  |

**References**

1. Kim YB, Okuda J, Matsumoto C, Takahashi N, Hashimoto S, Nishibuchi M: **Identification of Vibrio parahaemolyticus strains at the species level by PCR targeted to the toxR gene**. *J Clin Microbiol* 1999, **37**(4):1173-1177.

2. Venkateswaran K, Dohmoto N, Harayama S: **Cloning and nucleotide sequence of the gyrB gene of Vibrio parahaemolyticus and its application in detection of this pathogen in shrimp**. *Appl Environ Microbiol* 1998, **64**(2):681-687.

3. Luan XY, Chen JX, Zhang XH, Jia JT, Sun FR, Li Y: **Comparison of different primers for rapid detection of Vibrio parahaemolyticus using the polymerase chain reaction**. *Letters in applied microbiology* 2007, **44**(3):242-247.

4. Taniguchi H, Ohta H, Ogawa M, Mizuguchi Y: **Cloning and expression in Escherichia coli of Vibrio parahaemolyticus thermostable direct hemolysin and thermolabile hemolysin genes**. *J Bacteriol* 1985, **162**(2):510-515.

5. Taniguchi H, Hirano H, Kubomura S, Higashi K, Mizuguchi Y: **Comparison of the nucleotide sequences of the genes for the thermostable direct hemolysin and the thermolabile hemolysin from Vibrio parahaemolyticus**. *Microb Pathog* 1986, **1**(5):425-432.

6. Tada J, Ohashi T, Nishimura N, Shirasaki Y, Ozaki H, Fukushima S, Takano J, Nishibuchi M, Takeda Y: **Detection of the thermostable direct hemolysin gene (tdh) and the thermostable direct hemolysin-related hemolysin gene (trh) of Vibrio parahaemolyticus by polymerase chain reaction**. *Mol Cell Probes* 1992, **6**(6):477-487.

7. Nishibuchi M, Murakami A, Arita M, Jikuya H, Takano J, Honda T, Miwatani T: **Detection with synthetic oligonucleotide probes of nucleotide sequence variations in the genes encoding enterotoxins of Escherichia coli**. *J Clin Microbiol* 1989, **27**(10):2272-2276.

8. Matsumoto C, Okuda J, Ishibashi M, Iwanaga M, Garg P, Rammamurthy T, Wong HC, Depaola A, Kim YB, Albert MJ *et al*: **Pandemic spread of an O3:K6 clone of Vibrio parahaemolyticus and emergence of related strains evidenced by arbitrarily primed PCR and toxRS sequence analyses**. *J Clin Microbiol* 2000, **38**(2):578-585.

9. Okura M, Osawa R, Iguchi A, Takagi M, Arakawa E, Terajima J, Watanabe H: **PCR-based identification of pandemic group Vibrio parahaemolyticus with a novel group-specific primer pair**. *Microbiol Immunol* 2004, **48**(10):787-790.

10. Nasu H, Iida T, Sugahara T, Yamaichi Y, Park KS, Yokoyama K, Makino K, Shinagawa H, Honda T: **A filamentous phage associated with recent pandemic Vibrio parahaemolyticus O3:K6 strains**. *J Clin Microbiol* 2000, **38**(6):2156-2161.

11. Williams TL, Musser SM, Nordstrom JL, DePaola A, Monday SR: **Identification of a protein biomarker unique to the pandemic O3:K6 clone of Vibrio parahaemolyticus**. *J Clin Microbiol* 2004, **42**(4):1657-1665.
