# Supplementary material for: Genome plasticity of Vibrio parahaemolyticus: microevolution of the 'pandemic group'
Source: BMC Genomics. 2008 Nov 28;9:570. doi: 10.1186/1471-2164-9-570 (PMC2612023; doi:10.1186/1471-2164-9-570)
Supplement: Additional file 3 — Distribution of tdh and trh genes in the 174 strains. [file 1471-2164-9-570-S3.doc]

**Additional file 3. Distribution of *tdh* and *trh* genes**


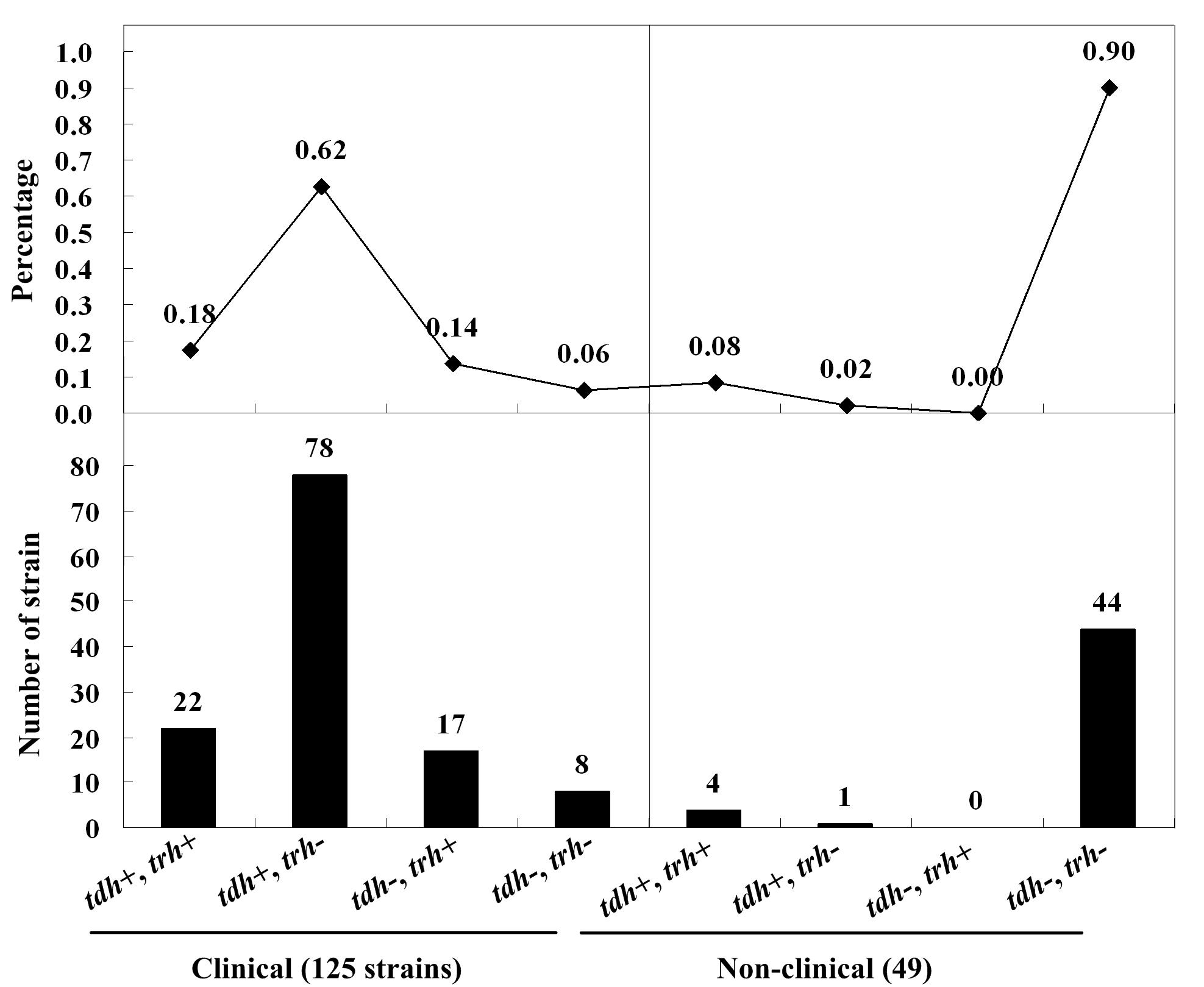


Strains were binned according to the presence (+) or absence (-) of *tdh* and *trh*. Bottom: number of strains in each bin. Top: percentage of strains in each bin in relative to the total clinical or non-clinical strains.
