# Supplementary material for: Genome plasticity of Vibrio parahaemolyticus: microevolution of the 'pandemic group'
Source: BMC Genomics. 2008 Nov 28;9:570. doi: 10.1186/1471-2164-9-570 (PMC2612023; doi:10.1186/1471-2164-9-570)
Supplement: Additional file 4 — The M-GCH data of representative strains, S008, S093, S133, S068, S087, S080, and S082. [file 1471-2164-9-570-S4.doc]

**Additional file 4. The M-GCH data of representative strains**


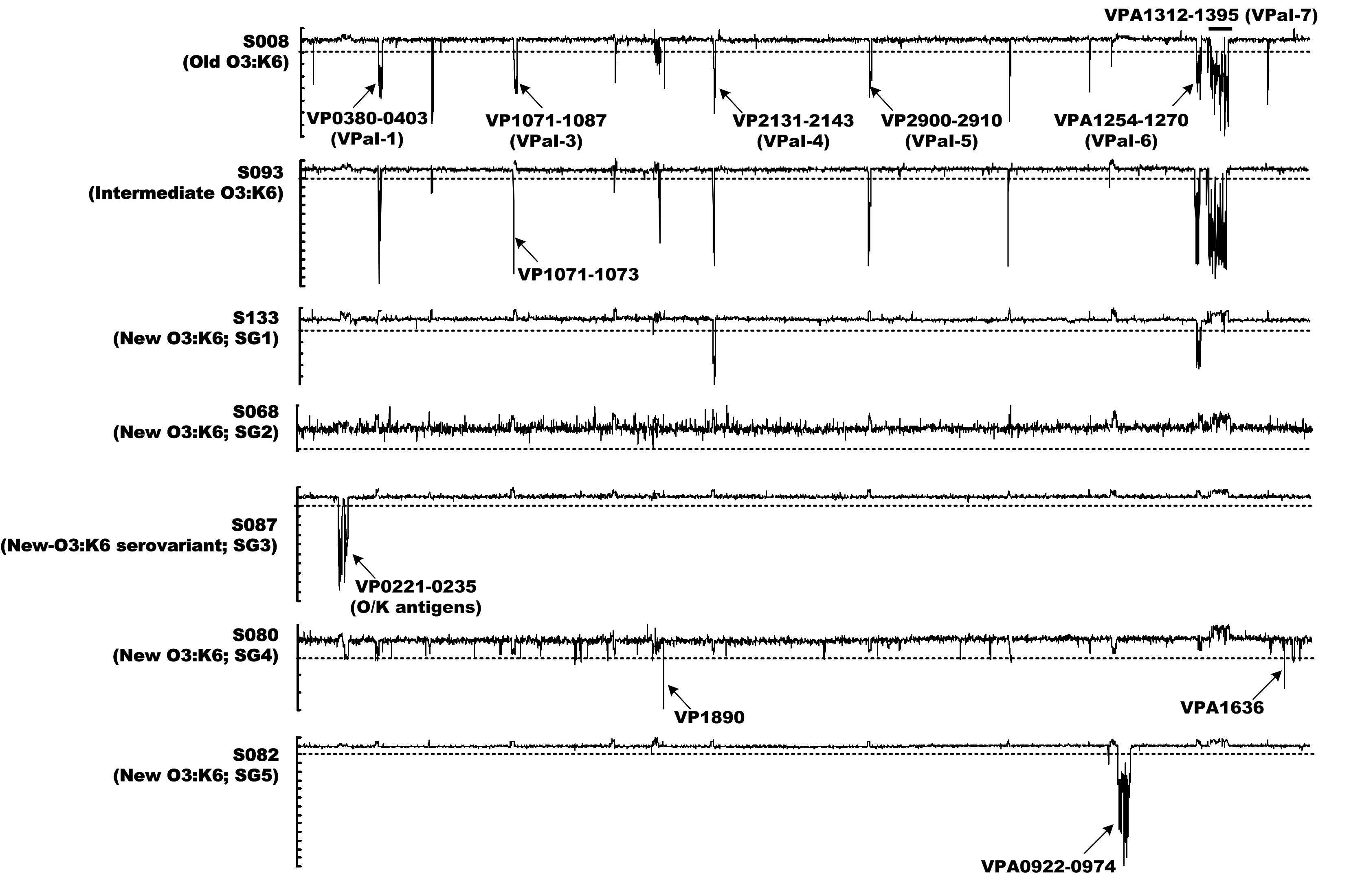


Genes are displayed on the X axis by the genomic location. The log_2_ hybridization ratio of each gene is displayed on the Y axis. Gene has a log_2_ ratio less than -1 (the dash line) indicated its absence from the indicated strain. Shown are selective genes encoding genomic islands and O/K antigens.
