# Supplementary material for: Genome plasticity of Vibrio parahaemolyticus: microevolution of the 'pandemic group'
Source: BMC Genomics. 2008 Nov 28;9:570. doi: 10.1186/1471-2164-9-570 (PMC2612023; doi:10.1186/1471-2164-9-570)
Supplement: Additional file 5 — Distribution of variably- and universally-present genes according to their GC composition. [file 1471-2164-9-570-S5.doc]

**Additional file 5. Distribution of variably- and universally-present genes according to their GC composition**

**
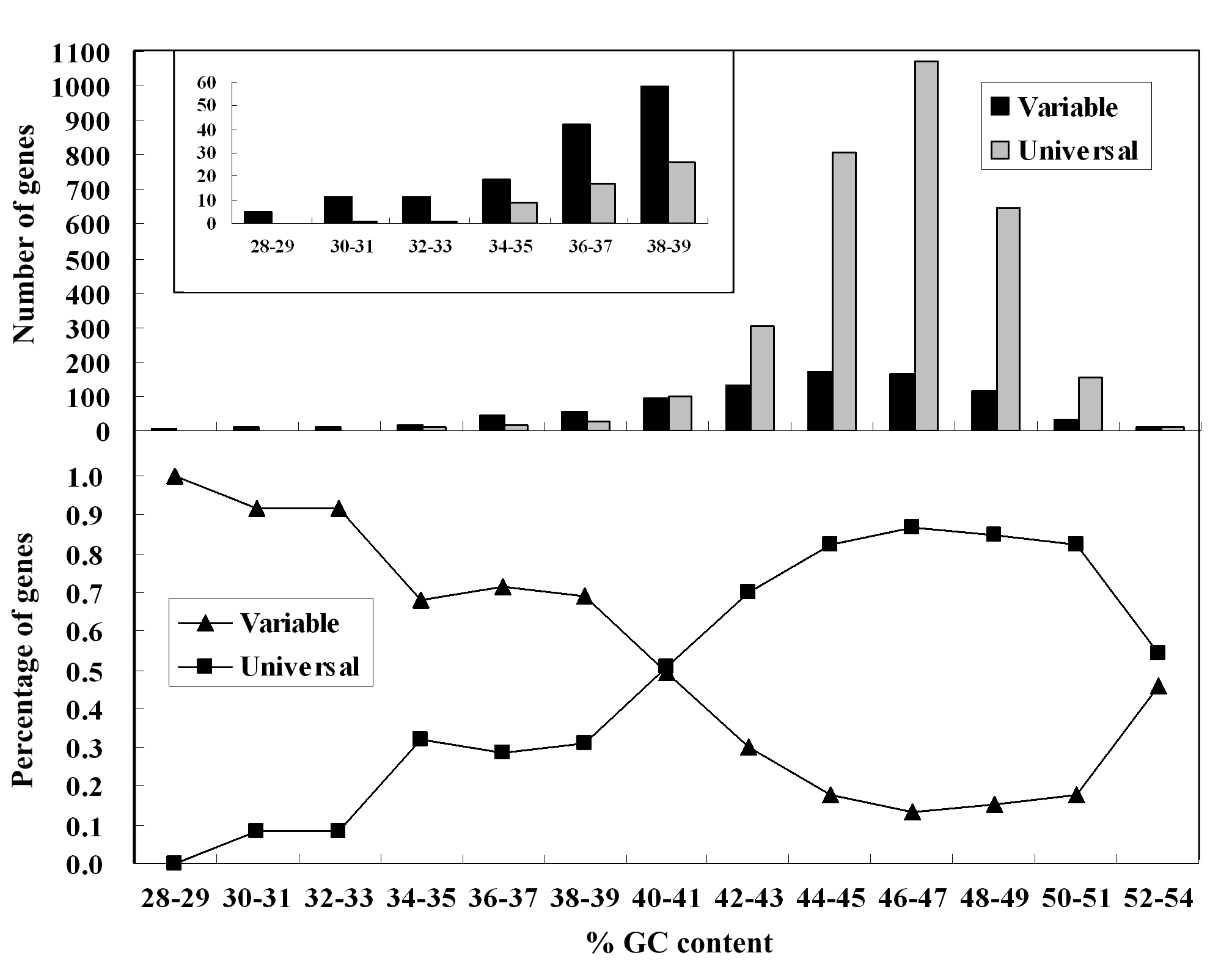
**

Genes were binned according to GC content in steps of 2%. Top: number of variably- and universally-present genes in each fraction (28-29, 30-31, etc) of GC content. Bottom: percentage of variably- and universally-present genes in each fraction of GC content.
