# Supplementary material for: Genome plasticity of Vibrio parahaemolyticus: microevolution of the 'pandemic group'
Source: BMC Genomics. 2008 Nov 28;9:570. doi: 10.1186/1471-2164-9-570 (PMC2612023; doi:10.1186/1471-2164-9-570)
Supplement: Additional file 6 — Distribution of variably-present genes according to their functional category. [file 1471-2164-9-570-S6.doc]

**Additional file 6. Distribution of variably-present genes according to their functional category**

**
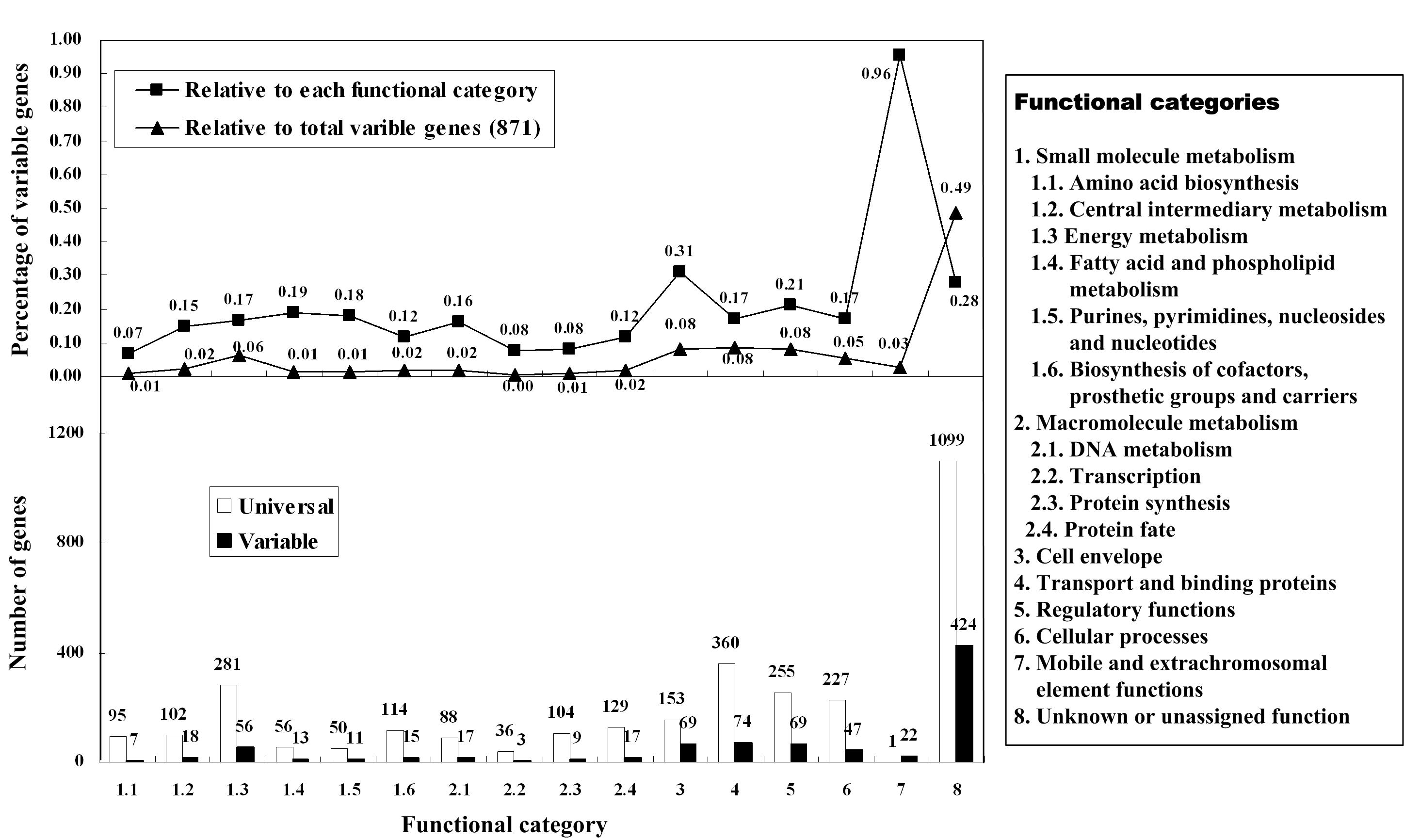
**

Genes were binned according to their functional categories. Bottom: number of variably- and universally-present genes in each functional category. Top: percentage of variably-present genes within each bin in relative to the total 871variably-present genes or to the total genes within each bin.
